# Mapping the Use of Ketamine in Treatment-Resistant Depression and Other Psychiatric Disorders: A Scoping Review of Practice Patterns, Efficacy, and Patient Demographic Trends

**DOI:** 10.1097/MJT.0000000000001951

**Published:** 2025-04-15

**Authors:** Amna M. Aslam, Kenneth Shinozuka, Owen Muir, Burton J. Tabaac

**Affiliations:** 1 Fermata Health, Brooklyn, NY;; 2 American Society of Ketamine Physicians, Psychotherapists, and Practitioners (ASKP), Austin, TX;; 3 Centre for Eudaimonia and Human Flourishing, University of Oxford, Oxford, United Kingdom;; 4 Department of Psychiatry, University of Oxford, Oxford, United Kingdom;; 5 Acacia Clinics, Sunnyvale, CA;; 6 Reno School of Medicine, University of Nevada, Reno, NV; and; 7 Department of Neurology, Carson Tahoe Health, Carson City, NV.

**Keywords:** ketamine, treatment-resistant depression, psychiatric care, scoping review, health care accessibility

## Abstract

**Background:**

Ketamine has emerged as a novel treatment for psychiatric disorders, particularly treatment-resistant depression (TRD). Although intravenous (IV) ketamine is not approved by the Food and Drug Administration (FDA) for TRD, esketamine, an FDA-approved therapeutic, has contributed to the widespread clinical use of off-label IV ketamine across the United States. This scoping review highlights significant shifts in ketamine therapy, particularly after FDA approval of esketamine, the impact of COVID-19 on treatment accessibility, and increased regulatory scrutiny from the Drug Enforcement Administration (DEA) and FDA.

**Areas of Uncertainty:**

What are the current practice patterns, patient demographics, and barriers to accessing ketamine for psychiatric disorders, particularly TRD?

**Data Sources:**

This scoping review focused on provider utilization patterns (including frequency of ketamine administration, provider roles, and treatment settings), preferred administration methods (IV infusions, intramuscular injections, and other routes), and patient characteristics (age, sex, socioeconomic status, and primary psychiatric diagnoses treated). The Web of Science, PubMed, CBM, MEDLINE, Cochrane Library, University Theses, and Embase databases were searched.

**Results:**

Two survey-based studies were included. IV administration was the most common method of administration reported in both studies, with alternative methods such as intramuscular and sublingual routes emerging in limited use. Patients receiving ketamine therapy were predominantly middle aged (36–64 years old), with financial barriers identified as a notable obstacle because of limited insurance coverage. Access to ketamine was limited in rural areas, illustrating the need for expanded provider networks. Private clinics exhibited greater flexibility in treatment approaches than hospital settings, which adhered to standardized protocols. The absence of long-term outcome data and variability in treatment protocols emphasize the need for standardized practices and further research.

**Conclusions:**

This scoping review highlights the widespread use of ketamine for TRD, but reveals significant variability in practice patterns and accessibility barriers. Findings emphasize the need for standardized protocols, expanded insurance coverage, and further research to optimize the role of ketamine in psychiatric care.

## INTRODUCTION

Major depressive disorder (MDD) is prevalent, affecting millions of people globally.^1,2^ Despite the availability of treatments such as electroconvulsive therapy, antidepressants, and transcranial magnetic stimulation, a considerable proportion of individuals with MDD do not achieve adequate relief, leading to treatment-resistant depression (TRD).^3^ TRD is characterized by the failure to achieve symptom remission after at least 2 trials of antidepressants from different pharmacologic classes at adequate doses and durations.

The prevalence of TRD is driving the exploration of novel treatments, such as ketamine, esketamine (an enantiomer of ketamine), and ketamine-assisted therapy.^3–8^ Ketamine, a dissociative anesthetic, has demonstrated rapid antidepressant effects in patients with TRD.^3,7^ Although a nasal spray formulation of esketamine was approved by the FDA in 2019, intravenous (IV) ketamine is not yet FDA approved for treating depression. As more evidence emerges of the efficacy of IV ketamine to address depression, its off-label use has become more commonly prescribed.^4,6,9^

This scoping review aims to map ketamine use for TRD, explore practice patterns and patient demographics, and identify barriers to access. By synthesizing these findings, readers are provided an overview of the role of ketamine in psychiatric care, and this review highlights gaps for future research and policy development opportunities.

## METHODS

**FIGURE 1.**
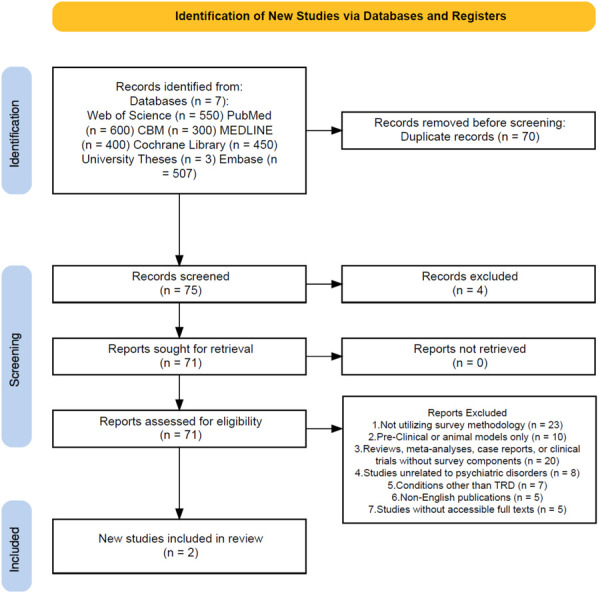
Literature search flowchart. The scoping review adhered to the Preferred Reporting Items for Systematic reviews and Meta-Analyses - Scoping Review (PRISMA-ScR) guidelines, and this flowchart was created from the PRISMA 2020 flow diagram template. A combination of manual and automated techniques was used to remove duplicate records. Screening resulted in the manual deletion of irrelevant articles. Two studies were identified that met our inclusion criteria.

For this scoping review, 2 reviewers independently selected studies from Web of Science, PubMed, CBM, MEDLINE, Cochrane Library, University Theses, and Embase (Figure 1). Titles and abstracts were screened, followed by full-text evaluations to confirm eligibility. Discrepancies were resolved through discussion and consensus, ensuring a thorough selection process.

The inclusion criteria focused on survey studies addressing ketamine use in psychiatric care, particularly TRD. Studies were excluded if they did not use a survey methodology, concentrated exclusively on preclinical or animal models, comprising reviews, meta-analyses, case reports, or clinical trials lacking a survey component. Further exclusions applied to studies unrelated to psychiatric disorders or publications in languages other than English. Studies without accessible full texts were also excluded from consideration. Eligible studies used survey-based methods to map clinical practices, provider perceptions, and operational characteristics. Data on ketamine administration methods, such as IV, intramuscular (IM), sublingual, and treatment protocols and dosages, were required.

Studies included needed to report outcomes related to TRD, including treatment efficacy (eg, symptom reduction, response rates), patient demographics (eg, age, sex, diagnosis history), safety practices (eg, monitoring protocols, adverse events), and financial elements (eg, insurance coverage, out-of-pocket expenses).

Two reviewers independently conducted data extraction to ensure accuracy and thoroughness. One reviewer performed the initial extraction, while another verified the data for consistency and completeness. Discrepancies were resolved through discussion and consensus among all reviewers, ensuring reliability.

The extraction process focused on key aspects of ketamine utilization in psychiatric care. Study design characteristics were documented, emphasizing the survey-based methodologies used in the included studies. Detailed information relevant to ketamine treatment practices was collected, including administration methods such as IV, IM, and sublingual routes, and reported dosages and treatment frequencies.

Data on patient demographics were extracted to provide insights into the populations receiving ketamine therapy, including age, sex, and socioeconomic backgrounds. The primary conditions treated with ketamine were noted (eg, TRD, MDD, and post-traumatic stress disorder (PTSD)). Provider roles encompassed physicians, psychiatrists, anesthesiologists, psychologists, nurses (RN, NP, LPN, CRNA), behavioral therapists, and other specified health care professionals. Treatment settings (private clinics or hospital-affiliated facilities) were also documented.

Economic factors, including the extent of insurance coverage and the prevalence of out-of-pocket payments, were assessed to highlight financial barriers to treatment. Information on reported side effects, such as dissociation and dizziness, was collected to evaluate treatment tolerability. In addition, outcome measures were included to assess patient improvement and operational practices.

Data from the included studies were synthesized thematically to provide an overview of ketamine use in psychiatric care, highlighting practice patterns, patient populations, and barriers to access. This methodology ensures a focused and reliable examination of ketamine's clinical and operational role. This review does not include clinical trials or meta-analyses assessing ketamine efficacy, which may limit comparisons of real-world practice patterns with controlled research outcomes.

## RESULTS

A sample of 2 studies met all of the inclusion criteria for this scoping review. Both studies used survey-based methodologies and focused on ketamine use in psychiatric care, specifically for TRD and other mental health conditions. The studies captured data from ketamine providers across diverse clinical settings, highlighting practice patterns, patient demographics, and barriers to access.

The conditions treated with ketamine included TRD (n = 2 studies), MDD (n = 2), PTSD (n = 1), and anxiety disorders (n = 1) (Table 1). Among providers surveyed, IV ketamine was the predominant administration route reported (n = 2), followed by IM and sublingual methods (n = 1 each). However, recently, providers have increasingly explored nonintravenous routes of administration as potential options for improving patient convenience and compliance (n = 1). These methods, although less common than IV ketamine, showed promise in broadening the applicability of treatment, particularly for patients with needle aversion or limited access to infusion clinics.

**Table 1.** Ketamine treatment landscape: clinical and operational insights.

Category	Key findings	Identified study
Disease treated	TRD (n = 2), MDD (n = 2), PTSD (n = 1), anxiety disorders (n = 1)	Wilkinson et al,^7^ Aslam et al^3^
Administration routes	IV (n = 2), IM (n = 1), sublingual (n = 1). Variability noted in preference	Wilkinson et al,^7^ Aslam et al^3^
Provider characteristics	Primarily outpatient clinics, led by psychiatrists (n = 2) and anesthesiologists (n = 1)	Wilkinson et al,^7^ Aslam et al^3^
Adverse effects	Common side effects: Dissociation (n = 2), dizziness (n = 1), transient blood pressure increase (n = 1)	Wilkinson et al,^7^ Aslam et al^3^
Financial barriers	Most patients pay out of pocket (n = 2), limited insurance reimbursement reported	Wilkinson et al,^7^ Aslam et al^3^
Geographic access	Urban areas have higher provider concentration. Rural areas face limited access (n = 2)	Wilkinson et al,^7^ Aslam et al^3^
Adjunctive treatments	Psychotherapy integrated with ketamine by some providers (n = 1), others used standalone	Aslam et al^3^
Dosing protocols	Dosing variability observed; research-backed protocols versus modified regimens (n = 2)	Wilkinson et al,^7^ Aslam et al^3^
Insurance coverage	FDA-approved esketamine shows better coverage than off-label IV ketamine (n = 1)	Aslam et al^3^
Trends in practices	Shift toward alternative methods such as sublingual and IM for accessibility (n = 2)	Wilkinson et al,^7^ Aslam et al^3^
Provider training	Limited training resources for providers, particularly nonpsychiatric professionals (n = 1)	Aslam et al^3^
Follow-up mechanisms	Few clinics track long-term outcomes; monitoring focuses on short-term effects (n = 2)	Wilkinson et al,^7^ Aslam et al^3^

Patient demographics indicated that ketamine treatments were predominantly accessed by middle-aged adults (36–64 years old) (n = 2), with 50% representation of male patients and 50% representation of female patients (n = 2). Socioeconomic data revealed that most patients belonged to higher income brackets (middle class $70,000–$105,999 and upper middle class $106,000–$140,999) (n = 2), reflecting significant financial barriers to care. Geographic disparities in access were noted, with urban centers housing a higher concentration of ketamine providers than rural areas (n = 2). This uneven distribution limited availability for patients in less densely populated regions, often requiring significant travel to reach treatment centers. Providers in rural settings reported additional challenges in obtaining resources and maintaining clinic sustainability because of lower patient volumes.

Barriers to access were identified as key challenges. Out-of-pocket payment was the primary payment model for most patients (n = 2), with economic constraints cited as a significant limitation to treatment availability. Some providers issued reimbursement for FDA-approved intranasal esketamine, whereas off-label IV ketamine was typically uninsured (n = 1). However, securing insurance coverage was often described as time intensive and uncertain, leading many patients to rely on self-payment. This financial dynamic shaped clinic operations, because some providers adjusted their pricing models to make treatments more accessible to patients. Providers often offered tiered pricing or installment plans to improve accessibility for patients unable to secure insurance reimbursement (n = 1). Despite these efforts, the high cost of treatment remained a barrier for many, contributing to disparities in care. One study noted that certain patient populations, particularly those from underserved areas, had limited access to ketamine clinics, compounding the issue of geographic inequity.

Providers also reported a lack of standardization in treatment protocols (n = 2), referring to inconsistencies in dosing schedules, administration routes, treatment frequency, patient monitoring practices, and criteria for patient eligibility. In addition, providers highlighted insufficient long-term data on safety and efficacy (n = 1). Although most clinicians adhered to research-backed dosing regimens, such as 0.5 mg/kg for IV infusions, most clinics reported modifying doses based on patient response or specific conditions being treated. These modifications were driven by a lack of universal guidelines, underscoring the need for standardized protocols to reduce variability in treatment outcomes.

Post-treatment outcomes were not consistently tracked across the studies, with providers emphasizing the need for additional research into adjunctive therapies, such as psychotherapy, to optimize patient outcomes. Monitoring practices varied, with most providers conducting basic physiologic checks during and after ketamine administration (n = 2). Provider characteristics revealed that most ketamine treatments were administered in outpatient clinic settings (n = 2). These clinics were typically independent practices rather than hospital-affiliated facilities, allowing for more flexibility in treatment protocols. Providers included psychiatrists (n = 2), anesthesiologists (n = 1), and other specialists with mental health or pain management expertise. The interdisciplinary nature of ketamine providers highlighted its diverse application across clinical domains (Table 1).

The surveys captured variability in adjunctive treatments offered alongside ketamine. Providers reported integrating psychotherapy with ketamine infusions in some cases (n = 1), while others focused solely on pharmacologic administration without concurrent mental health interventions (n = 1). The combination of ketamine and therapy was noted to enhance patient engagement and treatment outcomes, although the practice was not universally adopted.

Providers monitored adverse effects associated with ketamine treatment. Commonly reported side effects included dissociation (n = 2), dizziness (n = 1), and transient increases in blood pressure (n = 1). These effects were generally mild and managed effectively through routine monitoring protocols. Notably, 1 study emphasized the need for more robust guidelines to address rare but potentially severe complications, such as psychological distress postinfusion.

Provider feedback included a focus on the need for better education and resources. Some providers expressed concerns about limited access to training on ketamine administration, particularly for nonpsychiatric professionals entering the field. Wilkinson et al^7^ revealed that 33.3% of providers came from nonpsychiatric backgrounds, including specialties in anesthesiology (22.8%), emergency medicine (3.5%), and family medicine (3.5%). Similarly, Aslam et al’s^3^ study on practice patterns found that 44.12% of providers were general medical doctors, while only 30.88% were psychiatrists or psychologists, with the remaining 25% coming from various health care fields such as nurses and behavioral therapists. The lack of specialized training was perceived as a barrier to maintaining consistent safety and efficacy standards across clinics (Table 1).

The studies noted a lack of comprehensive follow-up data in many practices. Although providers typically monitored short-term patient responses during and immediately after treatment, fewer clinics maintained mechanisms to track long-term outcomes. This gap in longitudinal data was identified as a significant limitation for advancing ketamine research and developing evidence-based recommendations. Given the small sample of included studies (n = 2), findings may not fully capture the diversity of ketamine use across all clinical settings, highlighting the need for future large-scale analyses to enhance generalizability.

## DISCUSSION

### Main findings

This scoping review evaluated ketamine use in psychiatric care, with an emphasis on the application to TRD. Data from 2 comprehensive surveys provided insights into clinical practices, patient demographics, and operational challenges faced by providers. The findings underscore the widespread adoption of ketamine as a therapeutic option for individuals with TRD. IV ketamine was the most commonly reported route of administration, reflecting established efficacy and familiarity among providers.^5^ Alternative methods, including IM and sublingual administration, were noted but still need to be more frequently used.

The patient population most often receiving ketamine therapy is predominantly middle aged, with an equal distribution of male and female patients. Financial constraints were identified as a significant barrier, with most patients relying on out-of-pocket payments because of limited insurance coverage.^3^ This takeaway underscores the inequitable nature of ketamine access, with treatment often limited to patients with more economic resources. Providers reported limited progress in insurance reimbursement, even for FDA-approved formulations such as intranasal esketamine.

Practice patterns showed notable variability between clinical settings. Private clinics demonstrated greater adaptability in treatment protocols, with some integrating adjunctive therapies such as psychotherapy to enhance potential outcomes.^10^ In contrast, hospital-affiliated clinics adhered to more standardized practices, likely driven by institutional policies. These differences in approach reflect the broader diversity in ketamine delivery models, shaped by the organizational context of each setting.

The analysis also revealed geographic disparities in ketamine availability, with a higher density of providers in urban centers and limited access in rural regions. This disparate distribution restricts treatment options for underserved populations, emphasizing the need for expanded provider networks to improve accessibility. The studies also noted a lack of longitudinal data collection, with most providers focusing on short-term monitoring of physiologic and psychological responses. This gap limits the ability to assess the sustained efficacy and safety of ketamine in psychiatric care, underscoring the need for future research to address long-term outcomes.

Despite the growing use of ketamine therapy, long-term patient outcome data remain limited, underscoring the necessity for structured follow-up protocols to assess sustained efficacy and safety. The increasing presence of nonpsychiatric providers, including anesthesiologists and general practitioners, in ketamine therapy emphasizes the need for comprehensive training programs to ensure safe and effective administration of treatment. Expanding insurance coverage for off-label ketamine use through policy advocacy and reimbursement model adjustments could improve treatment accessibility for lower income patients. The onset of the COVID-19 pandemic in 2020 accelerated the adoption of at-home ketamine treatment models, prompting heightened scrutiny from regulatory bodies such as the DEA and FDA because of concerns about patient safety and prescribing practices. The increasing recognition of ketamine infusion therapy as an alternative to electroconvulsive therapy has contributed to improvements in insurance coverage, as demonstrated by recent comparative studies.^11^

Compared with Wilkinson et al,^7^ who found wide variability in dosing and administration protocols, Aslam et al^3^ report a shift toward more standardized infusion schedules and adjunctive therapeutic approaches. In addition, whereas Wilkinson et al^7^ reported that 64% of patients paid entirely out-of-pocket for ketamine treatment, before FDA approval, Aslam et al^3^ found that partial insurance coverage for FDA-approved esketamine had expanded access to a broader demographic.

In response to the growing demand for standardized practices, the American Society of Ketamine Physicians, Psychotherapists, and Practitioners launched a formal initiative in late 2023 to develop clinical guidelines aimed at improving safety and efficacy standards in ketamine treatment.

### Evolution of ketamine practices (2017 vs. 2024)

#### Adoption

At the time that Wilkinson et al^7^ was published, ketamine treatment for TRD was relatively new. The survey indicated that most providers had only started offering ketamine in the 5 years leading up to 2017. In the study by Aslam et al,^3^ ketamine treatment was more established. There was greater consensus on treatment protocols, with most clinics following standardized procedures for ketamine infusions, patient monitoring, and adjunctive treatments. Differences in efficacy, patient safety, and long-term results between the 2 studies partly stem from this evolving practice environment.

#### Insurance coverage and accessibility

Wilkinson et al^7^ discovered that 64% of patients needed to pay for ketamine treatment out of pocket, with minimal insurance reimbursements, making this treatment less accessible to the general population. In comparison, Aslam et al^3^ reported that more clinics are available to provide ketamine with at least partial insurance coverage, particularly for FDA-approved intranasal esketamine. This significant change has expanded access to a diverse population nationwide, especially individuals from lower socioeconomic backgrounds.

#### Regulatory and safety considerations

Wilkinson et al^7^ discussed concerns about safety and the potential for ketamine abuse because of a lack of data on the long-term effects of repeated dosing. Because the FDA had not yet approved ketamine to treat depression in 2017, providers offering ketamine treatment at the time operated in a regulatory gray zone. Clinics and physicians were required to use their discretion in determining how to administer ketamine, and there were no standard protocols to follow. This is reflected in the findings published by Wilkinson et al, where wide variability in dosing, monitoring, and treatment regimens was observed. However, Aslam et al^3^ reported a significant change in the regulation of ketamine use, specifically in light of FDA approval of intranasal esketamine for TRD. Providers have clearer guidelines on how to safely administer ketamine, monitor patients, and manage potential adverse events and side effects, which leads to more consistent patient outcomes. The regulatory advancements between 2017 and 2024 have facilitated the development of best practices for ketamine administration, including the standardization of dosing (eg, 0.5 mg/kg IV for 40 minutes) and the integration of safety measures such as blood pressure and heart rate monitoring during infusions.

In addition, the Risk Evaluation and Mitigation Strategies program established for esketamine has indirectly influenced how clinics manage off-label ketamine use. Although IV ketamine does not fall under the Risk Evaluation and Mitigation Strategies requirements, many clinics have adopted similar practices, such as close monitoring during administration and postinfusion observation periods. This standardization has improved patient safety and reduced the risks associated with ketamine treatments.

#### Adjunctive therapies and multimodal approaches

Wilkinson et al^7^ present ketamine primarily as a standalone treatment, with limited adjunctive therapies in conjunction with ketamine infusions. Aslam et al^3^ report a higher rate of clinics offering psychotherapy, medication management, and integration coaching with ketamine treatments. These additions result in significantly better patient outcomes.

## CONCLUSIONS

This scoping review highlights the widespread use of ketamine for TRD, with IV administration as the most commonly applied approach. Alternative routes, such as IM and sublingual, show potential but require further validation. Financial barriers, including limited insurance coverage, restrict access, particularly for lower income populations. Practice patterns vary between private clinics and hospital-affiliated settings, reflecting differences in flexibility and standardization.^10^ Geographic disparities further limit availability in rural areas. Addressing these challenges through standardized protocols, expanded insurance coverage, and improved accessibility is essential to optimize the role of ketamine in psychiatric care.
